# Tilapia Fish Skin Treatment of Third-Degree Skin Burns in Murine Model

**DOI:** 10.3390/jfb14100512

**Published:** 2023-10-11

**Authors:** Carissa Garrity, Christina Garcia-Rovetta, Iris Rivas, Ubaldo Delatorre, Alice Wong, Dietmar Kültz, Jamie Peyton, Boaz Arzi, Natalia Vapniarsky

**Affiliations:** 1 Department of Pathology, Microbiology, and Immunology, University of California, Davis, CA 95616, USA; crgarrity@ucdavis.edu (C.G.); irlrivas@ucdavis.edu (I.R.);; 2 Department of Anatomy, Physiology and Cell Biology, School of Veterinary Medicine, University of California, Davis, CA 95616, USA; 3 Department of Animal Sciences and Coastal & Marine Sciences Institute, Davis, CA 95616, USA; dkueltz@ucdavis.edu; 4 One Health Institute, School of Veterinary Medicine, University of California, Davis, CA 95616, USA; jlpeytondvm@icloud.com; 5 Department of Surgical and Radiological Sciences, School of Veterinary Medicine, University of California, Davis, CA 95616, USA; barzi@ucdavis.edu

**Keywords:** wound regeneration, burn therapy, fish skin, hematology, histology

## Abstract

This study explored the feasibility of using fish skin bandages as a therapeutic option for third-degree skin burns. Following the California wildfires, clinical observations of animals with third-degree skin burns demonstrated increased comfort levels and reduced pain when treated with tilapia fish skin. Despite the promises of this therapy, there are few studies explaining the healing mechanisms behind the application of tilapia fish skin. In this study, mice with third-degree burns were treated with either a hydrocolloid adhesive bandage (control) (*n* = 16) or fish skin (*n* = 16) 7 days post-burn. Mice were subjected to histologic, hematologic, molecular, and gross evaluation at days 7, 16, and 28 post-burn. The fish skin offered no benefit to overall wound closure compared to hydrocolloids. Additionally, we detected no difference between fish skin and control treatments in regard to hypermetabolism or hematologic values. However, the fish skin groups exhibited 2 times more vascularization and 2 times higher expression of antimicrobial defensin peptide in comparison to controls. Proteomic analysis of the fish skin revealed the presence of antimicrobial peptides. Collectively, these data suggest that fish skin can serve as an innovative and cost-effective therapeutic alternative for burn victims to facilitate vascularization and reduce bacterial infection.

## 1. Introduction

Severe skin burns are painful and may be life-threatening injuries, posing a substantial public health concern. Worldwide, an estimated 180,000 deaths occur annually due to burns, with the majority occurring in low- and middle-income countries [1]. Non-fatal burn injuries are among the leading causes of morbidity [1]. Treatment of burns typically requires prolonged hospitalization and may result in disfigurement and disability, often with resulting stigma and rejection. In the United States alone, it is estimated that approximately 359,000 people sought medical care for burns in 2020 [2]. Global warming-associated wildfires and the associated burn victims are increasing worldwide at an alarming rate [3]. These statistics associated with burns invite the development of novel, affordable therapies for burn patients.

Burns can be categorized depending on the degree of damage inflicted. First-degree burns are defined as superficial burns consisting of damage only to the epidermis. These burns usually heal within seven days of injury. Second-degree burns involve the epidermis and dermis and can be further categorized into superficial partial thickness (epidermis and papillary dermis) or deep partial thickness (epidermis and reticular dermis). Superficial partial thickness burns typically heal within 10 days and require debridement of damaged skin and daily wound care. Deep partial-thickness burns have a more variable healing time, usually between 10 and 28 days, and require surgical excision and resurfacing in addition to daily wound care. A burn that affects the entire dermis is categorized as third degree, will require surgical excision and resurfacing, and typically heals within three weeks or more. Once a burn reaches tissues deep in the dermis, such as muscle, fat, and bone, it is categorized as a fourth-degree burn and often requires amputation and more extensive reconstruction [4].

Burns injuries are not just limited to skin and can result in systemic complications. Burns exceeding 30% of total body surface area can trigger a systemic inflammatory response, thus resulting in hypovolemic burn shock, a hypercoagulable state, and a risk of sepsis [5]. Furthermore, the extreme metabolic challenge associated with severe burns results in hypermetabolism syndrome. The exact pathophysiology is still elusive, but it has been recognized as a marked increase in catecholamines and cortisol and changes in acute phase protein pathways, leading to increased metabolic rates, systemic increases in catabolism, and multi-organ dysfunction [6]. Hew et al. demonstrated that post-burn hypermetabolism is induced in mice with both large (10% TBSA) and small (2.8% TBSA) burns using parameters such as basal energy expenditure, changes in catabolism, and changes in hormones and inflammatory mediators [7]. These alterations in metabolism and inflammation may impede the healing process of wounds and introduce new complications that necessitate medical attention.

Substantial progress in burn care has significantly improved the survival of burn victims [4]. However, more improvements can be achieved in the area of skin replacement. For example, autologous skin grafting may be currently considered the gold standard for burn treatment, but this option is not suitable for patients with extensive burns and has the disadvantage of inducing morbidity at the collection site [8]. Biological and synthetic skin substitutes offer various advantages; however, at this juncture, there is no ideal skin substitute on the market that provides effective, affordable, and scar-free wound healing [9]. Currently, available treatment options may leave patients at risk of complications such as pain, scarring, and infection, underscoring the need for medical advancements in skin-replacement modalities.

Tilapia skin has been explored as a promising burn treatment since it was first used in a clinical trial on humans in Brazil in 2017 [10]. According to Hu et al., 99.14% of peptides extracted from tilapia skin by hydrolysis were less than 5 kDa, with 58% hydrophilic residues. These peptides were effective in reducing the scratch defects of keratinocytes as effectively as an epidermal growth factor. Also, the wound-healing rate in the rabbit in vivo model treated with marine peptides was enhanced compared to burn ointment and untreated control groups [11]. The abundance and relatively uncomplicated farming of Nile tilapia, combined with its low cost, makes tilapia skin an attractive solution for burn therapy. The affordability of tilapia skin is especially relevant for low-income societies where burn injuries are more prevalent. Furthermore, recent wildfire events in California led to a large number of burn victims among wild and domesticated animals. With a lack of affordable skin-replacement options, tilapia skin was used to treat extensive third-degree burn wounds in these animals. Although no controlled studies were performed, the clinical observations pointed out a marked reduction in pain and an increase in comfort levels of tilapia skin-treated animals. These clinical observations inspired this study, aiming to explore the efficacy of tilapia fish skin bandages for full-thickness burns in mice compared to hydrocolloid bandage-treated controls. Here, we report a comparison of wound sizes, complete blood counts, body weights, histology, various molecular markers, and serum glucose levels between experimental groups. Additionally, we report proteomic analyses of nonstructural peptides of fish and murine skin.

## 2. Materials and Methods

### 2.1. Animals

For this experiment, 32 C57BL/6 mice with ages ranging from 12 to 15 weeks were divided into two groups where males and females were equally represented. This study was conducted with the approval of the Institutional Animal Care and Use Committee.

### 2.2. Study Design

**Figure 1:**
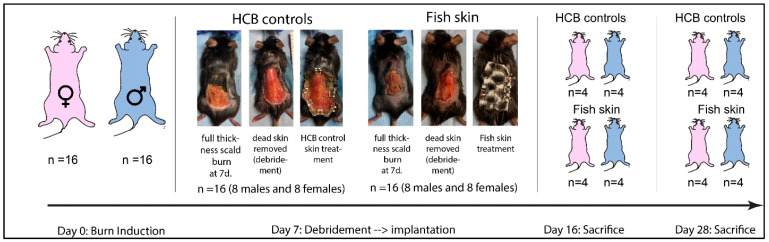
Study design and timeline of the study. A scald burn was induced on the dorsum. On day 7, the burned tissue was surgically debrided, and the wound was dressed in either a hydrocolloid bandage (HCB) or rehydrated fish skin. Both bandages were secured to the intact skin with metal clips. On day 16 and day 28, animals were humanely euthanized for tissue collection. The blood was drawn from the infraorbital sinus under anesthesia at days 0, 7, 16, and 28.

In all mice, scald burn was created on the dorsum at day zero. Body weights were recorded, and blood was collected into heparin and serum tubes. On day seven post-burn induction, the necrotic skin was surgically debrided, and the exposed wound was covered by either fish skin, hydrocolloid bandage (HCB), or adhesive bandage (control) (DuoDERM Cat # 187660) (Figure 1). Half of the mice in fish-skin-treated and HCB controls were humanely euthanized on day 16, while the remainder of the mice were euthanized on day 28 post-burn induction. Prior to euthanasia, blood was collected as before. Upon euthanasia, the fish skin and HCB bandages were removed, the wounds photographed, and the body weights of the animals recorded.

### 2.3. Burn Induction

The mice were anesthetized using isoflurane (0.5–2%) and oxygen (2 L/min) in an induction chamber. During the induction, buprenorphine (0.05 mg/kg SC), Ceftiofur (20 mg/kg SC), and warm saline (5 mL SC) were administered subcutaneously. The mice were then transferred to a table, and maintenance isoflurane anesthesia was delivered via a nose cone. Once no pain reflex was registered on the paw pinch, tetracaine eye drops were applied to each eye, and a heparin-treated hematocrit capillary tube was inserted into the retro-orbital sinus to withdraw blood. Four drops of blood were collected into serum and EDTA tubes, respectively. The blood was kept on ice until processing within a few hours. A complete blood count (CBC) was performed at UC Davis Comparative Pathology Laboratory, certified for murine blood analysis. Both eyes were then lubricated with ointment to prevent corneal drying during the anesthesia. The dorsum was shaved with beard clippers to expose approximately 2 cm × 3 cm area of skin. Veet hair removal cream was applied to the shaved area for 60 s to remove the hair completely. Once the cream was removed, each mouse was laid onto the plastic bed with the 20 × 30 mm opening (corresponding to 3–5% of total body surface area) overlapping the shaved area of skin. The scald burns were induced according to a previously published protocol with minor modifications [12]. Specifically, the water temperature was raised to 56 °C, and the exposure time was increased to 30 s to ensure induction of third-degree burns. Mice were in individual cages after the burn induction. Fluids and buprenorphine were administered prior to burn induction and then twice daily for the three days following burn induction. The mice were monitored daily for 10 days post-burn.

Although the burn-induction conditions were identical for all mice, smaller females with body weights of <25 g did not tolerate the burn procedure well and had to be euthanized before they reached the termination time points. For this reason, the study was repeated on additional mice with the best possible attempt to have a balanced sex representation across groups. The eventual numbers of mice across groups are presented in the results in Section 3.7.

### 2.4. Burn Debridement and Treatment

On the seventh day post-burn, the mice were weighed, anesthetized, and had blood collected via the same procedure outlined above. Once blood was collected, the wounded area of each mouse was surgically debrided, and fish skin or HCB (control) was applied to completely cover the wound (Supplementary Materials Video S1). Each dressing was secured to the surrounding skin with metal clips.

### 2.5. Sacrifice and Sample Collection

On the day of sacrifice, mice were anesthetized, and blood was drawn as previously described, but no fluids or other medications were administered. Upon sacrifice, the clips were removed before weighing. Anesthetized animals were placed into a CO_2_ chamber until no rib movement was observed. Post mortem, the skin samples from the burn-healthy skin interface were harvested and preserved frozen or fixed in formalin for further RNA isolation, proteomic analysis, and histology.

### 2.6. Fish Skin Preparation

The fish skin bandages were produced at UC Davis using a patented protocol https://patents.google.com/patent/US11612675B1 (accessed on 20 August 2023) [13]. This protocol is approved and publicly available. Briefly, whole tilapia fish were cleaned from scales and celomic contents. The skin was removed from the very fresh tilapia fish (<2 h post-mortem) by traction from the rostral-dorsal aspect of the fish body towards the tail using the pincher tool (Supplementary Materials Figure S1). The removed skin was placed in the ice bath for 10 min to prevent deterioration while processing other fish. The myofascial tissue was then thoroughly scraped away with a blunt knife or a hard scraper. After that, the skins were rinsed in a new container with sterile 0.9% saline and then transferred to the container containing 2% Chlorhexidine gluconate (VetOne 30159, VetOne, Boise, ID, USA) at 1 mL solution per each 1 cm^2^ of skin. The skins were soaked in this solution with gentle agitation for 30 min before a repeated rinse with saline. The process of Chlorhexidine gluconate soak and rinse was repeated one more time before the skins were transferred into 50% glycerol solution in saline for 1 h soak at room temperature with gentle agitation. Subsequently, the skins were soaked in 75% and 99% glycerol (Thermo 17904, Thermo Fisher Scientific, Waltham, MA, USA) for 1 h at room temperature and then 24 h at 4 °C, respectively. After that, the skins were preserved in 100% glycerol with 1% pen strep for up to three months.

Before application to the wounds, the skins were rehydrated in three subsequent soaks of sterile 0.9% saline for 20 min each. Once rehydrated, the skins were used in their entirety.

### 2.7. Wound Size

Photographs were taken on the day of debridement and the day of sacrifice once the bandages had been removed. Photographs were analyzed with Image J software (Java 1.8.0_172 (64-bit), National Institutes of Health, Bethesda, MD, USA), where the total area of the burn was measured at the time of debridement and upon sacrifice to yield the closure index (Closure index = ([wound size at Day 7] − [wound size at day 16/28]/[wound size on day 7]). Thus, a closure index value of one is indicative of complete closure, while a value of zero indicates a complete absence of wound closure. The closure indexes were compared between control and fish skin bandage-treated groups at two time points (day 16 and day 28).

### 2.8. Blood Analyses

Heparinized blood was submitted for complete blood count, while serum was analyzed for glucose.

### 2.9. PCR Testing

Total RNA was extracted from mouse skin using TRIzol (Invitrogen, Waltham, MA, USA) following the manufacturer’s protocol, and cDNA was generated using the QuantiTect Reverse Transcription Kit (Qiagen, Venlo, The Netherlands). RNA expression of genes listed in Table 1 was determined by real-time quantitative RT-PCR (Qiagen, Venlo, The Netherlands) using Qiagen’s QuantiNova SYBR Green PCR buffer. Each reaction was carried out in duplicates. Cycle conditions were denaturation at 95 °C for 2 min, followed by 40 cycles of denaturation at 95 °C for 5 s and annealing/extension at 60 °C for 10 s. The final melting curve step was performed according to Bio-Rad’s CFX 96 (Bio-Rad Laboratories, Hercules, CA, USA). The level of expression of the target gene was normalized to GAPDH. All calculations were formulated using the delta-delta-CT method. Primer sets were designed from known murine gene sequences or generated from previously published data (Table 1).

### 2.10. Proteomics

Skin samples were analyzed after tryptic in-solution digestion. Briefly, proteomics samples were collected, processed, and analyzed with LCMS using nanoAcquity (Waters, Milford, MA, USA) and Impact II (Bruker Daltonics, Billerica, MA, USA), as previously published [23,24,25]. Sequences of identified peptides were searched against mice and tilapia with PEAKS X Plus (BSI, Inc., Waterloo, ON, Canada) against Uniprot proteomes (UP000005207, UP000000589) to identify proteins present in samples. Identified proteins were then blasted against NCBI (https://blast.ncbi.nlm.nih.gov/Blast.cgi, accessed on 8 July 2020) using their six-digit accession number.

The complete set of proteomic and all other raw data is provided in a ( Supplementary Materials Table S1) and Public Data Repository (https://data.mendeley.com/datasets/hssv66g4kb/4, accessed on 14 September 2023). We screened and compared the peptides between murine and fish skin, created a new list of peptides that were present in the fish skin but not murine skin, and excluded structural peptides in both species. After the elimination of structural or Identical peptides from both species, the list was reduced to uncharacterized nonstructural peptides. Their sequences were then blasted against the mammalian protein database. Only proteins with > 90% identity to uncharacterized peptides in fish skin are reported here with STRING Consortium Version 11.5 (STRING, Zurich, Switzerland).

### 2.11. Histology

Initial histological evaluation was performed on hematoxylin and eosin-stained sections, where the extent of granulation tissue, presence and severity of inflammation, type of inflammatory cells, presence of bacterial contamination, and degree of re-epithelialization were assessed. Subsequently, snapshot images of the granulation tissue at the lesion interface at 20× and 200× magnification were acquired. The vascularization in the granulation tissue was then quantified via manual count with ImageJ on 200× magnification images. The quantification of vessels was normalized to the area of the section. Additional 5 µm sections were generated from the same paraffin blocks for immunohistochemical evaluation. Immunoreactivity for Factor VIII was applied in order to more precisely highlight and quantify the degree of neovascularization. Similarly, using 20× magnification images of the burn section snapshots, the thicknesses of the skin were measured at the burned–healthy skin interface location with ImageJ.

### 2.12. Immunohistochemistry (IHC)

All samples were sectioned at 5 µm. Briefly, FFPE sections were deparaffinized in xylene and hydrated in 100% ethanol before blocking endogenous peroxidase activity by incubating sections in 0.3% hydrogen peroxide in methanol for 30 min. Sections were then hydrated in decreasing concentrations of ethanol (95% and 70%, respectively). Enzymatic antigen retrieval was performed with Proteinase K (200 ug/mL) for 10 min at room temperature (RT) (Millipore IHC Select, Millipore, Louisville, KY, USA). Next, sections were blocked at (RT) with 10% horse serum in phosphate-buffered saline and Tween-20 (PBST) for 30 min. Sections were incubated with Polyclonal Rabbit Anti-Human von Willebrand Factor [1:2000] (Dako, A0082, Santa Clara, CA, USA) diluted in PBST for one hour at RT. One-step antigen detection was performed with Rabbit-on-Canine HRP-polymer (Biocare Medical, RC542, Concord, CA, USA) incubation for 30 min at RT. Vector NovaRed^®^ Peroxidase (HRP) Substrate Kit (Vector Laboratories, SK, 4800, Newark, CA, USA) was used to detect the antigen in murine skin samples. The specimens were then counterstained with Mayer’s Hematoxylin Solution (Sigma-Aldrich, MHS16, Youngstown, OH, USA) and washed in tap water to blue the stain. Specimens were dehydrated with a quick dip in 100% ethanol and xylene and coverslipped using a mounting medium (Vector labs Cat # H-5000-60, Detroit, MI, USA). Histological images were analyzed and captured on Olympus BX46 (EVIDENT, Bethlehem, PA, USA) equipped with a color camera Olympus DP 27. All images were captured at 20× magnification. The vasculature counts were performed on captured images with ImageJ.

### 2.13. Statistical Analysis

Statistics were created using GraphPad Prism version 9.5.1 (GraphPad Software, Dotmatics, Boston, MA, USA). All data presented are expressed as means with standard error. All data were checked for normality and transformed accordingly. Two-way ANOVA tests were used to compare control and fish-skin-treated mice at all time points in our PCR data and complete blood count evaluation. A paired *t*-test was used to compare fish-skin-treated groups to controls in CBC data, wound closure index, wound area, serum glucose, and body weight. All data were analyzed further to explore differences between male and female mice post-burn. Paired *t*-tests were used to compare male and female mice for each time point and parameter. Results were considered statistically significant at *p* < 0.05.

## 3. Results

### 3.1. Wound Size Evaluation and Quantification of Mice Treated with Fish Skin

**Figure 2:**
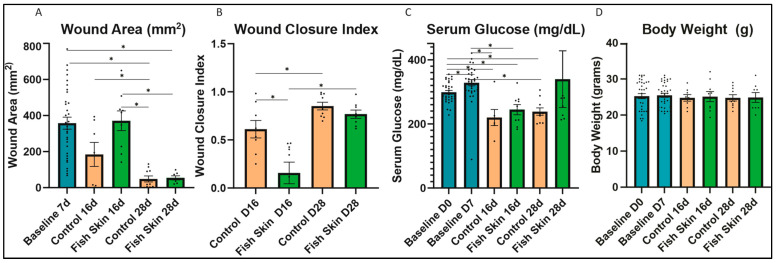
Wound healing and metabolism. (**A**) Wound area on day 7, day 16, and day 28. (**B**) The wound closure index was calculated with the following formula: (Closure index = ([wound size at Day 7] − [wound size at day 16/28]/[wound size at day 7]. (**C**) Body weight at days 0, 7, 16, and 28. (**D**) Serum glucose at days 0, 7, 16, and 28. Normal serum levels are indicated by the gray box. Asterisks annotation: *p* ≤ 0.05 (*). Baseline groups represent the average parameters of all mice, regardless of treatment. All mice were treated the same until day 7.

The wound size was measured on day 7 and on day 16 or day 28 (Figure 2A). The average percent of the total body surface area burned on day 7 was between 3 and 5%. Significant differences were detected in the wound closure indexes (Figure 2B). As mentioned earlier, a wound closure index of one indicates a full wound closure, while an index of zero indicates no wound closure. Fish skin treatment showed no benefit in wound closure over the controls at the 16-day time point. Specifically, the closure index was significantly higher in control mice than in the fish-skin-treated mice at 16 days. However, at the 28-day time-point, there was no significant difference in wound closure indexes between the groups. In both control mice and fish-skin-treated mice, there was a significant increase in the wound closure index at the 28-day time point relative to the 16-day time point.

### 3.2. Serum Glucose

We detected no significant differences in glucose levels between treatment groups at any time point in this study. On day 16, both HCB and fish-skin-treated mice showed a significant decrease in glucose relative to days 0 and 7 baseline values (Figure 2C). On day 28, control mice continued to have significantly lower than day 0 and day 7 glucose levels; in contrast, fish-skin-treated mice returned to the original value range.

### 3.3. Evaluation of Body Weight Changes

All mice, HCB and fish-skin-treated, showed no significant changes in body weight at any time point (Figure 2D). None of the test individuals dropped their body weight below the baseline range.

### 3.4. Complete Blood Count Evaluation

#### 3.4.1. White Blood Cells

**Figure 3:**
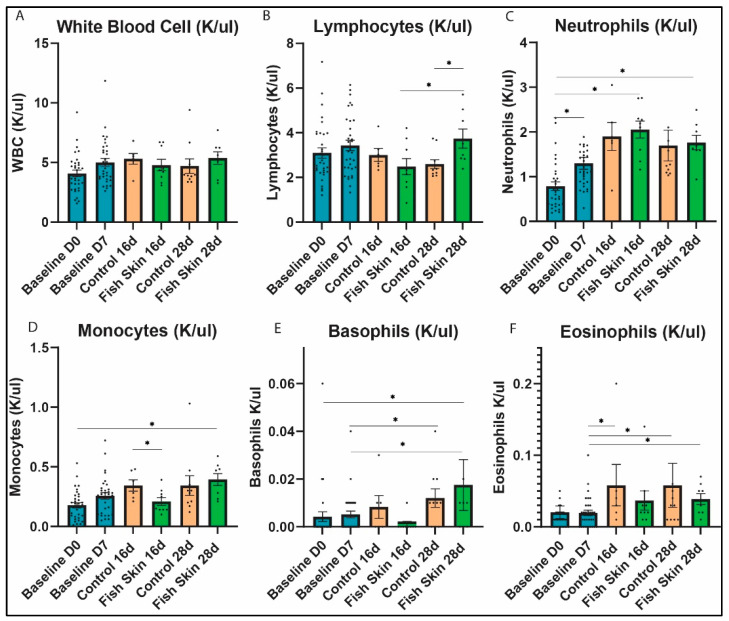
Complete leukocyte blood count analysis. (**A**) White blood cell count on days 0, 7, 16, and 28. (**B**) Lymphocyte counts on days 0, 7, 16, and 28. (**C**) Neutrophil counts on days 0, 7, 16, and 28. (**D**) Monocyte counts on days 0, 7, 16, and 28. (**E**) Basophil counts on days 0, 7, 16, and 28. (**F**) Eosinophil counts on days 0, 7, 16, and 28. Asterisks annotation: *p* ≤ 0.05 (*). Baseline groups represent the average parameters of all mice, regardless of treatment. All mice were treated the same until day 7.

All mice, controls and fish-skin-treated, showed no significant changes in total white blood cell (WBC) count at any time point (Figure 3A). Control mice showed a minimal increase in WBC count on day 16 and then a slightly lower WBC count on day 28. In contrast, fish-skin-treated mice showed virtually no change in counts on days 16 and 28.

#### 3.4.2. Lymphocytes

Absolute lymphocyte counts were significantly lower in the controls than in fish-skin-treated mice at the 28 d time point (Figure 3B). Although insignificant, an opposite trend was observed between the groups at the 16 d time point. Fish-skin-treated mice had significantly higher counts on day 28 relative to day 16. Data on lymphocyte percentages, provided in the Supplementary Materials (Figure S2A), generally follow the trend discussed above.

#### 3.4.3. Neutrophils

Both groups showed similar trends in absolute neutrophil counts (Figure 3C) as well as neutrophil percentages (Figure S2B), and we detected no significant differences in neutrophil counts or percentages between control and fish-skin-treated groups at any time points. Mice treated with fish skin had significantly higher neutrophil counts on day 16 and day 28 relative to day 0. At 28 days, fish-skin-treated mice showed an insignificant reduction in neutrophil absolute counts relative to day 16. Neutrophil counts of HCB-treated mice, although insignificant, showed an increase at day 16 and day 28 relative to baseline values. However, neutrophil counts of HCB-treated mice decreased at day 28 relative to day 16.

#### 3.4.4. Monocytes

Both groups showed similar trends of monocyte populations throughout the study. Absolute count (Figure 3D) and percentage (Figure S2C) of monocytes trended higher in HCB than fish skin groups except at day 28. However, the only significant difference between the control and fish skin groups was that monocyte counts were higher in controls at day 16. Control mice showed no significant difference in monocyte counts between 16 d and 28 d time points. In contrast, fish-skin-treated mice showed a significant increase in the number of monocytes on day 28 relative to day 0. Both groups showed a progressive increase in monocyte counts among the time points relative to the day 0 baseline.

#### 3.4.5. Basophils

All mice, HCB and fish-skin-treated, showed no significant differences in basophil percentages (Figure S2D) at any time point. However, basophil counts (Figure 3E) showed significant changes relative to baseline values in both groups. Fish-skin-treated mice showed a significant increase in basophil count on day 28 relative to day 7 and day 0. In contrast, HCB-treated mice only showed a significant change on day 28 relative to day 7.

#### 3.4.6. Eosinophils

Both groups showed similar trends in absolute eosinophil counts (Figure 3F) as well as eosinophil percentages (Figure S2E), and we detected no significant differences in eosinophil counts or percentages between control and fish-skin-treated groups at any time point. Eosinophil percentage and absolute counts trended higher in controls relative to fish skin. Mice treated with fish skin showed a significant increase in the percentage of eosinophils on day 16 and day 28 relative to day 7 (Figure S2E). Similarly, mice treated with fish skin showed a significant increase in eosinophil counts on day 28 relative to day 7. HCB groups also showed a significant increase in the percentage and number of eosinophils on day 16 and day 28 relative to day 7. Overall, no significant effect of fish skin treatments was found in eosinophil populations relative to controls.

#### 3.4.7. Red Blood Cell (RBC) Counts

**Figure 4:**
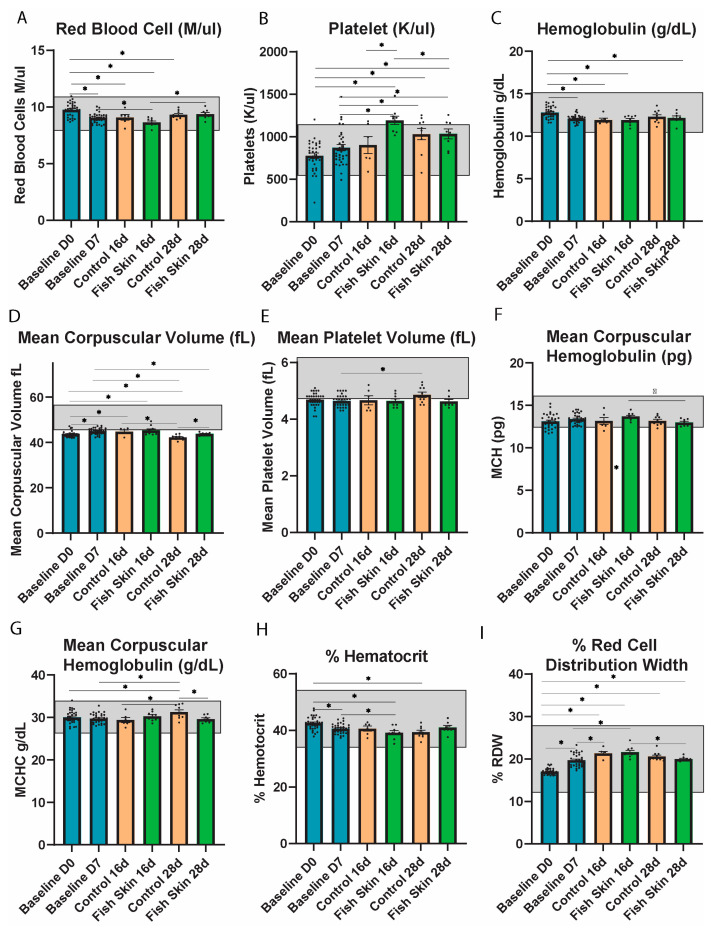
Complete blood parameter analysis. (**A**) Red blood cell count on days 0, 7, 16, and 28. (**B**) Platelet cell count at days 0, 7, 16, and 28. (**C**) Hemoglobulin concentration on days 0, 7, 16, and 28. (**D**) Mean corpuscular volume on days 0, 7, 16, and 28. (**E**) Mean platelet volume on days 0, 7, 16, and 28. (**F**) Mean corpuscular concentration on days 0, 7, 16, and 28. (**G**) Mean corpuscular hemoglobulin on days 0, 7, 16, and 28. (**H**) Percent hematocrit on days 0, 7, 16, and 28. (**I**) Percent red blood cell distribution width on days 0, 7, 16, and 28. Asterisks annotation: *p* ≤ 0.05 (*). Baseline groups took the average of all mice, regardless of treatment. All mice were treated the same until day 7.

Both fish skin and control mice showed similar trends and no significant differences in RBC counts at any time point (Figure 4A). In both groups, RBC counts were below the baseline at 16 d time points. At 28 d, the values returned to the 7 d baseline range in both groups. The RBC counts decreased significantly in the fish-skin-treated group between days 7 and 16, but not in controls.

#### 3.4.8. Platelets

The platelet counts followed the opposite trend of RBC counts and demonstrated an expected increase between day zero and days 7, 16, and 28 (Figure 4B). Fish-skin-treated mice showed a significant increase in platelet counts at day 16 and day 28 relative to both baseline time points. In contrast, HCB-treated mice only showed a significant increase in platelet counts on day 28 relative to both baseline time points. Platelet counts showed a significant difference between HCB and fish-skin-treated mice on day 16. However, this trend dissipated at day 28 when the platelet counts were almost identical between the study groups.

#### 3.4.9. Hemoglobulin

Both groups had a decrease in hemoglobulin levels on day 16 relative to baseline values (Figure 4C). Hemoglobulin levels increased minimally on day 28 relative to day 16 in both groups, but there were no significant differences between the groups at any time point of the study.

#### 3.4.10. Mean Corpuscular Volume (MCV)

HCB and fish-skin-treated mice showed opposite trends with regard to the mean corpuscular volume (MCV) of red blood cells (Figure 4D). MCV values in both groups were almost identical to 7 d baseline values. However, on day 28, both groups had a decrease relative to day 16 and 7 time points. Fish-skin-treated mice showed significantly lower and closer to the baseline MCV value relative to the control at day 28.

#### 3.4.11. Mean Platelet Volume (MPV)

MPV had a significantly higher value in controls on day 16 relative to day 7. Otherwise, no significant difference was recorded between treatment groups at any time point in the study (Figure 4E).

#### 3.4.12. Mean Corpuscular Hemoglobulin (MCH)

HCB groups maintained a normal and consistent level of mean corpuscular hemoglobulin (MCH) throughout the study (Figure 4F). MCH values of fish-skin-treated mice remained identical to baseline groups on day 16 but then significantly decreased on day 28 relative to day 16. In contrast, MCH values of HCB-treated mice, although insignificant, remained lower than baseline values. Overall, there were no significant differences between the control and fish skin groups at any time point.

#### 3.4.13. Mean Corpuscular Hemoglobulin Concentration (MCHC)

Fish-skin-treated mice maintained a consistent level of MCHC similar to baseline values throughout the study (Figure 4G). MCHC values of HCB groups decreased slightly initially but then showed a significant increase on day 28 relative to day 7 and relative to day 16. Additionally, the MCHC of HCB groups on day 28 was significantly higher than fish skin groups on day 28.

#### 3.4.14. Percent of Hematocrit

Both fish skin and control groups showed similar trends of hematocrit throughout the study. On day 16, hematocrit values of fish skin trended lower than controls (Figure 4H). This trend was reversed at day 28 when hematocrit values of fish skin groups increased and surpassed the values of control groups. Fish skin groups showed significant decreases on day 16 relative to day 7 and day 0. Similarly, hematocrit values of HCB groups decreased significantly on day 28 relative to day 0. Control groups maintained a fairly consistent level for the remainder of the study. There were no significant differences between fish skin and control groups.

#### 3.4.15. Percent Red Blood Cell Distribution Width (RDW)

Both fish skin and control groups showed similar trends of increasing RDW at all time points. There were no significant changes between the control and fish skin groups (Figure 4I). However, fish skin groups suggested higher rates of change between different time points in comparison to control groups. For example, both groups had a significant increase in RDW values on day 16 relative to day 7 and day 0.

### 3.5. Histology

**Figure 5:**
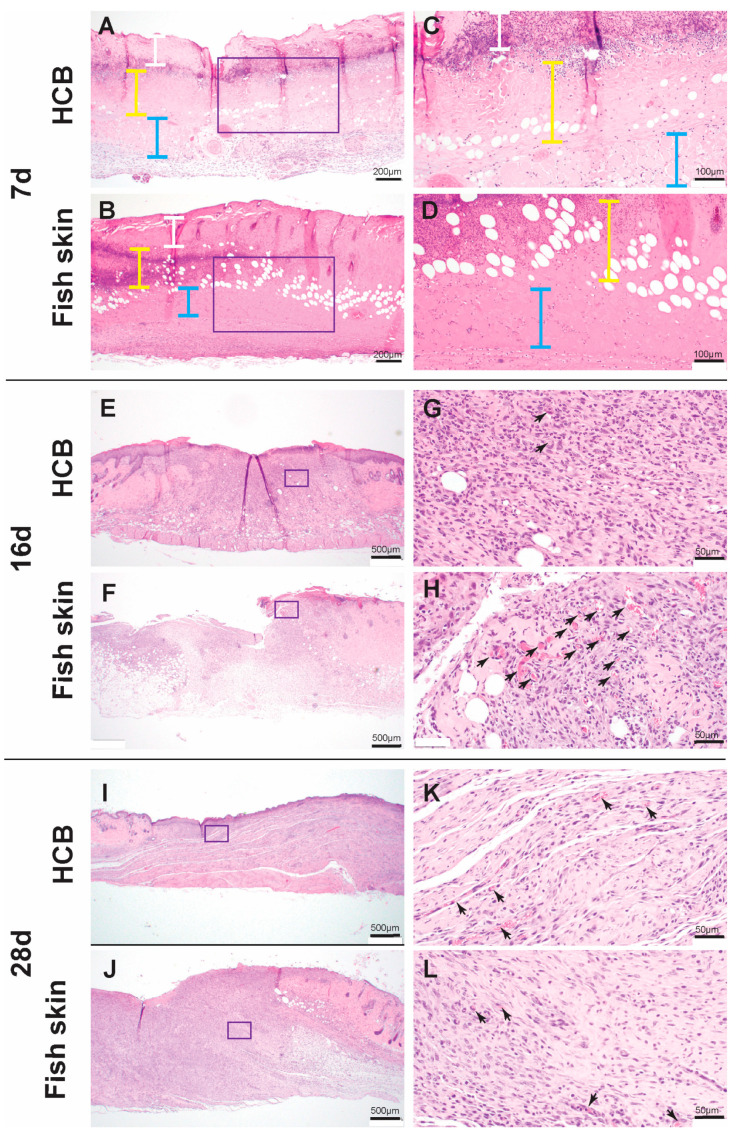
Histology. (**A**–**D**): Low- (**A**,**B**) and high- (**C**,**D**) magnification images of murine skin seven days post-burn induction. High-magnification snapshots represent areas highlighted by purple rectangles. Note full-thickness coagulative necrosis of epidermis and dermis (white vertical line), hypodermis (yellow vertical line), and panniculus carnosus (blue vertical line). The coagulative necrosis is characterized by diffuse eosinophilia and loss of cellular and nucellar detail. Subtending the necrotic dermis, there is a band of degenerate leucocytes (predominantly neutrophils). (**E**–**H**): Low- (**E**,**F**) and high- (**G**,**H**) magnification of murine skin 16 days post-burn induction. High-magnification snapshots represent areas highlighted by purple rectangles. Newly formed capillaries (angiogenesis) are indicated by black arrows. Note more pronounced angiogenesis in the granulation tissue areas of the fish-skin-treated mice. (**I**–**L**): Low- (**I**,**J**) and high- (**K**,**L**) magnification images of murine skin 28 days post-burn induction. High-magnification snapshots represent areas highlighted by purple rectangles. Note angiogenesis is almost equivalent in the granulation tissue in controls and fish-skin-treated mice (black arrows).

Histology confirmed the presence of full-thickness skin burns in all mice (Figure 5). Sections collected on day 7 (during the debridement procedure) contained denatured epidermis, dermis, hypodermis, hair follicles, and panniculus carnosus. The coagulative necrosis affecting these structures was characterized by a loss of cellular detail, sprinkling of nuclear fragments, and uniform eosinophilia. These layers formed a thick crust layered with abundant fibrin, degenerate neutrophils, cellular debris, fragmented hair shafts, and extravasated RBCs.

Sections collected on day 16 had markedly thickened and hypercellular hypodermis, especially prominent at the lesion interface with the normal skin. The hypercellularity was contributed by abundant fibroblasts and inflammatory cells (histiocytes, neutrophils, lymphocytes, and occasional mast cells) supported by a loose, at times, myxomatous matrix. Multiple small-caliber blood vessels and capillaries were abundant in these locations (angiogenesis). When quantified by the manual count using ImageJ, mice treated with fish skin had more pronounced angiogenesis than controls, albeit the difference was not found to be statistically significant. Interestingly, and consistent with the clinical observation of skin contraction around the burn, the panniculus carnosus layer extended farther than the epidermis towards the lesion center. Some re-epithelialization was observed at the lesion interface with the healthy skin, where a thickened epidermal layer of the intact skin was contiguous with a thin (1–2 cell thick) layer of neo-epidermis covering the granulation tissue formed instead of hypodermis and dermis. The skin thickness at the interface of the burn wound with healthy skin was not significantly different between the treatment groups at the 16 d time point, but the fish-skin-treated group trended slightly higher than the control. No new hair follicle formation was observed in the regenerating skin regions in any of the treatment groups.

Skin sections from the 28 d time point had a similar appearance to that described for 16 d with the exception of a much smaller wound size and, in some cases, almost complete re-epithelialization of the granulation tissues bed. No new hair follicle formation was observed in the regenerating skin regions in any of the treatment groups. Some regenerative changes were observed in the carnosus muscle. These included the rowing of myocyte nuclei and the formation of the thin, slightly basophilic in color myofibers. Quantification of angiogenesis on HE sections detected a higher but statistically insignificant trend in fish-skin-treated individuals. This trend was reversed on day 28. The skin thickness at the interface with healthy skin was significantly higher in fish-skin-treated mice at this time point (Figure S3).

### 3.6. Immunohistochemistry

**Figure 6:**
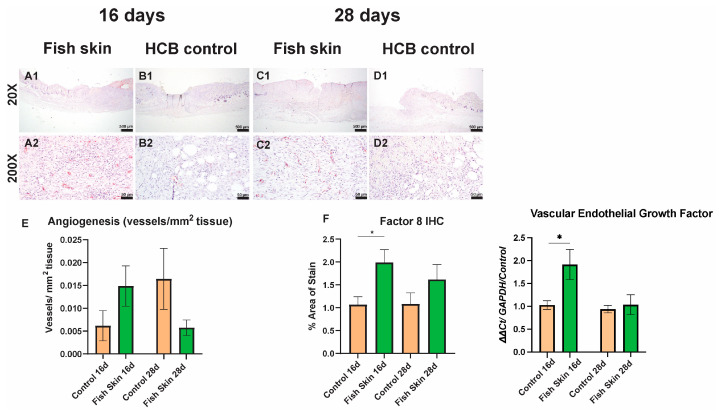
Quantification of angiogenesis. (**A1**,**C1**) and (**A2**,**C2**) micro-captures represent low and high magnification images of Factor 8-immunolabeled histological section of skin from the burn-healthy skin interface in fish skin-treated mice. (**B1**,**D1**) and (**B2**,**D2**) micro-captures represent low and high magnification images of immunolabeled histological section of skin from the burn-healthy skin interface.in mice treated with HCB. (**E**) Blood vessel formation was quantified with ImageJ on H&E-stained histology sections. (**F**) Blood vessel formation was quantified with ImageJ on Factor 8-immunolabeled sections. All counts were normalized to the area of the section. Asterisks annotation: *p* ≤ 0.05 (*).

Immunolabeling for factor 8 revealed a significant increase in the number of vessels in the fish-skin-treated mice at the 16 d time point. A similar trend, albeit statistically insignificant, was detected on day 28 (Figure 6).

### 3.7. Survival Outcomes

The burn induction had a detrimental effect on some of the smaller mice (females with a body weight of 20 to 23 g), and as a result, some losses were encountered. Specifically, these mice exhibited hunched body posture and tremors, refused to eat and drink, and had increased skin turgor, indicative of dehydration. Due to these reasons, the decision for humane euthanasia was made. Due to these losses, we repeated the study on additional animals to keep a sufficient representation of males and females across groups. Table 2 represents the final counts and distribution of males and females among treatment groups. Nonetheless, no statistically significant differences were observed between males and females for any outcome parameters examined. 

### 3.8. PCR Results

**Figure 7:**
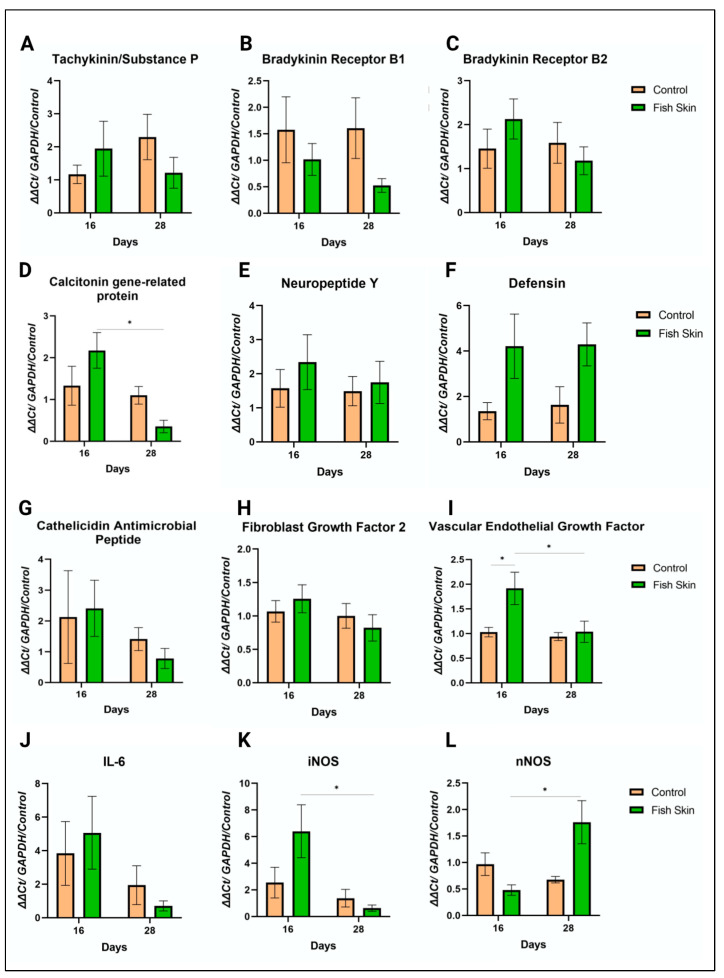
Gene expression analysis with QRT-PCR. The ΔΔCt values for each gene are calculated by averaging duplicate runs and normalizing the expression of the gene to GAPDH and control mice. (**A**) Gene Expression of Tachykinin/Substance P at day 16 and day 28 of control and fish skin groups normalized to controls and GAPDH expression. (**B**) Gene Expression of Bradykinin receptor B1 at day 16 and day 28 of control and fish skin groups normalized to controls and GAPDH expression. (**C**) Gene Expression of Bradykinin receptor B2 at day 16 and day 28 of control and fish skin groups normalized to controls and GAPDH expression. (**D**) Gene Expression of Calcitonin gene-related protein at day 16 and day 28 of control and fish skin groups normalized to controls and GAPDH expression. (**E**) Gene Expression of Neuropeptide Y at day 16 and day 28 of control and fish skin groups normalized to controls and GAPDH expression. (**F**) Gene Expression of Defensin at day 16 and day 28 of control and fish skin groups normalized to controls and GAPDH expression. (**G**) Gene Expression of Cathelicidin Antimicrobial Peptide at day 16 and day 28 of control and fish skin groups normalized to controls and GAPDH expression. (**H**) Gene Expression of Fibroblast Growth Factor 2 at day 16 and day 28 of control and fish skin groups normalized to controls and GAPDH expression. (**I**) Gene Expression of Vascular Endothelial Growth Factor at day 16 and day 28 of control and fish skin groups normalized to controls and GAPDH expression. (**J**) Gene Expression of Interlukin-6 at day 16 and day 28 of control and fish skin groups normalized to controls and GAPDH expression. (**K**) Gene Expression of inducible nitric oxide synthase at day 16 and day 28 of control and fish skin groups normalized to controls and GAPDH expression. (**L**) Gene Expression of neuronal nitric oxide synthase at day 16 and day 28 of control and fish skin groups normalized to controls and GAPDH expression. Asterisks annotation: *p* ≤ 0.05 (*).

PCR analysis investigated the expression of several genes encoding for pain perception, antimicrobial peptides, wound healing-associated proteins, and angiogenesis. Substance P (SubP) is a neuropeptide responsible for pain perception and the recruitment of early responders of inflammation [26]. The expression of this gene was reversed between the fish skin and control groups (Figure 7A). Specifically, on day 16, SubP expression trended higher in the fish skin group than in controls, while on day 28, this trend was reversed. Overall, the fish skin group had a trend of decreased SubP expression with respect to study time points, while the opposite was detected in controls. However, neither of the differences was found to be statistically significant. Similar trends were observed for bradykinin receptors B1 and B2 (Figure 7B,C). Bradykinin Receptors B1 and B2 (BKRB1 and BKRB2) are G-protein coupled receptors that bind bradykinin. Bradykinin peptides are responsible for eliciting many responses, such as vasodilation, edema, pain fiber stimulation, and inflammation [27,28,29]. BKRB1 expression trended higher in control groups at both time points. The expression of BKRB2 had the opposite trend among treatment groups. Specifically, on day 16, BKRB2 expression trended higher in fish skin than in control groups, while on day 28, this trend was reversed. Neither of the differences was found to be statistically significant. Calcitonin gene-related protein (CGRP) is another neuropeptide responsible for pain transmission and vasodilation [30,31]. CGRP expression trended similarly to BKRB2 (Figure 7D). The expression of neuropeptide Y (NPY), another stress response neuropeptide, trended higher in the fish skin group than controls at all time points (Figure 7E). However, similarly to BKRB1 and BKRB2, fish-skin-treated mice exhibited decreased expression of these genes over the study’s time points, while controls tended to remain unchanged.

Gene expression of defensin (Figure 7F) and cathelicidin antimicrobial peptide (CAMP) (Figure 7G) was performed to assess if fish skin treatment offers any benefit in protection against bacterial infections [32]. The fish skin group had 2.5 times more defensin expression than controls on day 16. Defensin expression increased slightly in control groups on day 28 but was still lower than in fish-skin-treated mice. However, CAMP expression did not follow similar trends. CAMP expression was minimally higher in the fish skin group than in controls on day 16, and this trend was reversed on day 28. No significant differences were observed between fish skin and control groups in either defensin or CAMP expression.

The expression of fibroblast growth factor 2 (FGF2) (Figure 7H) was investigated for its important role in wound healing. Fibroblast growth factor β (FGF2) is a signaling protein responsible for initiating tissue repair, cell growth, and sometimes vascularization [33,34]. At day 16, FGF2 expression in the fish skin group trended higher than in controls, while this trend was reversed at day 28. The expression of FGF2 decreased in the fish skin group over study time points, whereas the control group stayed principally unchanged. No significant differences between fish skin and control groups were detected at any time points.

The expression of vascular endothelial growth factor (VEGF) gene was carried out for its central role in angiogenesis. Fish-skin-treated mice had nearly two times more VEGF expression than control mice on day 16 (Figure 7I). On day 28, fish-skin-treated mice showed a significant decrease in VEGF relative to day 16 that leveled out to values similar, yet still higher, than HCB groups.

The expression of interleukin-6 (IL-6), neuronal (n), and inducible isoform (i) nitric oxide synthases (NOS) was investigated for their important role in inflammation, vascularization, stress, and insulin resistance. On day 16, IL-6 expression in fish-skin-treated mice trended higher than in controls, while this trend was reversed on day 28 (Figure 7J). The expression of IL-6 decreased in both groups over the study’s time points. Similar trends were observed with the expression of iNOS (Figure 7K). These trends were reversed for the expression of nNOS (Figure 7L). Collectively, no significant differences between fish skin and control groups were detected at any time points.

### 3.9. Proteomics

**Figure 8:**
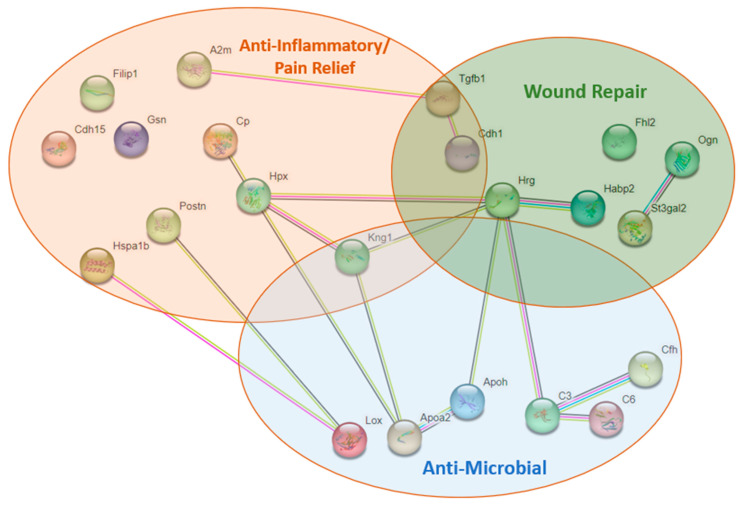
Proteomic analysis. Proteomic analysis revealed 22 nonstructural proteins unique to fish skin. A2M = alpha-2-macroglobulin; FILP1 = Filamin A Interacting Protein 1; CDH15 = Cadherin 15; GSN = gelsolin; CP = Ceruloplasmin; HPX = Hemopexin; POSTN = periostin; HSPA1B = Heat shock protein 1B; KNG1 = Kiniogen 1; CDH1 = Cadherin 1; TGFB = Transforming growth factor beta; HRG = Histidine-rich glycoprotein; FHL2 = four and a half LIM; HBP2 = hyaluronan-binding protein; OGN = osteoglycin; ST3GAL2 = CMP-N-acetylneuraminate-beta-galactosaamine-alpha-2,3-sialyltransferase; APOH = Apolipoprotein; C6 = Complement 6; APOA2 = Apolipoprotein-A; CFH = Complement factor-H; C3 = Complement 3; LOX-protein-lysine 6-oxidase.

After removing cell-structure-related (actins, keratins, etc.) proteins and extracellular matrix-related proteins (collagens) from the list of total peptides identified in fish and murine skin samples, we found 22 uncharacterized peptides in the fish skin that were not detected in the murine skin. Among these proteins, 11 had up to 90% sequence similarity with mammalian anti-inflammatory or pain relief peptides, 7 with wound/tissue repair peptides, and 7 with antimicrobial peptides (Figure 8). Some proteins had overlapping functions in each of the groups listed.

## 4. Discussion

This study is the first to evaluate the effects of fish skin burn treatment using histological, hematological, and molecular methods in a comprehensive and controlled fashion in a murine model. We also performed a proteomic comparison between mammalian and fish skin and identified unique proteins with anti-inflammatory, wound-repair, and antimicrobial properties in fish skin. Burn treatment with fish skin offered no benefit compared to hydrocolloid treatment in wound closure. We detected no difference between fish skin and HCB treatment regarding hematological values or systemic glucose concentration. However, we found that fish-skin-treated mice had increased angiogenesis at the earlier time point than the HCB-treated controls. We also detected a trend of higher antimicrobial peptide expression in fish-skin-treated mice compared to controls. Collectively, these findings provide insight into the main drivers behind fish skin therapy for burns and provide directions for further investigation.

Wound closure is an important indicator of therapeutic efficacy. Accelerating wound healing is ideal for a faster return to function. Contrary to our hypothesis and the published report by Ibrahim et al. [35], who showed a decrease in the percentage of overall wound size over 3 weeks in donkey metacarpal wounds treated with fish skin vs. untreated controls, our study detected no benefit of fish skin therapy on wound closure (Figure 2). This disagreement can be due to the difference in experimental design and animal species. Specifically, donkeys belonging to the family of Equidae heal their wound in a fashion similar to humans, i.e., by re-epithelializing the granulation tissue bed forming at the wound site [36]. In contrast to Equidae and humans, mice heal their wounds by contracting the dermis-associated panniculus carnosus muscle [37,38,39]. In our experiments, we observed that shortly after application, the fish skin bandages became hard shell-like structures, while HCB bandages remained soft and pliable throughout the course of the study. As a result, HCB-treated burn wounds contracted more efficiently, and their size reduction was more rapid than fish-skin-treated wounds. In other words, fish-skin treatment acted similarly to Dunn’s wound-splinting model [38], while HCB treatment did not, rendering it not the most appropriate control treatment for this study. It is possible that repeating this study using a large animal model such as a pig [40] or using a splinting approach would be more appropriate to evaluate the wound-closure effects of fish skin burn wound treatment.

We attempted to determine if mice in our study had evidence of hypermetabolic syndrome. However, the steady body weight and lack of hyperglycemia suggest that hypermetabolic syndrome did not occur in this study (Figure 2). Hypermetabolic syndrome usually occurs in cases with more extensive burns comprising TBSA of 60% or more [41]. In the current study, the % TBSA affected by burn ranged between 3 and 5% and was probably insufficient to cause hypermetabolic syndrome. The fluctuations in glucose values thus likely reflect a stress response rather than hypermetabolism syndrome. Also, an increase in IL-6 was reported to be associated with hypermetabolism syndrome in pediatric patients [42,43]. Specifically, a marked increase relative to pre-burn values was reported around day 7, with a subsequent decrease of approximately 1.5 to 2-fold at days 16 and 28, respectively [42], with a bio-plex ELISA assay. In our study, a similar decrease in IL-6 expression was detected, but it remains to be determined if RNA expression also correlated with the protein levels. In concert, more studies and probably a more extensive burn area would be needed to determine the effects of fish skin therapy on hypermetabolic syndrome.

Leukograms from the mice showed no evidence of infection or excessive consumption (Figure 3). In comparison with reports from human studies [44,45], neutrophil counts had similar fluctuations in our study, most likely reflecting the expected response to extensive tissue damage. Further studies evaluating the neutrophils’ specific phenotypes and secretome patterns [46] are essential to identify the potential benefits of fish skin therapy for burns. Monocytes, like neutrophils, are also considered one of the first responders to arrive at the site of burn injury during the early inflammatory phase. Interestingly, we detected significantly fewer systemic monocytes in fish-skin-treated mice than in controls on day 16. On day 28, the monocyte counts were similar between the treatment groups, and counts trended higher than on day 16 or at the baseline, but not substantially (Figure 3). Histopathological evaluation of H&E sections of the burn sites revealed no difference in macrophage or neutrophil infiltration between groups, but this assessment is subjective and requires more detailed investigation. It is important to investigate if fish skin treatment has an effect on macrophage polarization to types one and two [47]. M1 macrophages usually secrete high levels of iNOS and IL-6 [48,49]. Our PCR data demonstrated that iNOS and IL-6 expression was higher in fish-skin-treated skin samples than in HCB controls on day 16. On day 28, these trends were reversed, and IL-6 and iNOS expression were higher in HCB than in the fish-skin-treated group. These data indirectly suggest that fish skin treatment may be more inductive for the M1 macrophage phenotype at the earlier time points than the HCB control. These results align with studies by Guo et al., who found that burn injury increased the expression of iNOS but not nNOS [50]. Immunohistochemical studies are needed to assert this assumption.

We identified significantly higher lymphocyte counts in the fish-skin-treated mice than in HCB controls at the 28 d time point (Figure 3). This observation is intriguing and deserves a deeper investigation. Specifically, provided the fish skin is a xenogeneic tissue, it may induce an immune response in the recipient mice. A further look into specific lymphocyte phenotypes at the wound site and local and systemic cytokine profiles would be required to answer this question.

Fluctuations in red blood cell values in this study are numerous, but all primarily reflect an expected response to minimal blood loss [51], with no significant differences identified between the treatment groups. According to Duncan et al., reticulocytes (i.e., newly formed RBC) have a larger MCV and lower hemoglobin concentration. Hence, baseline MCV combined with a lower-than-baseline MCHC represents regenerative erythropoiesis [51]. Our study revealed a significant decrease in RBC counts on day 16, followed by an increase on day 28 in both groups. The latter changes most likely represent marginal blood loss. This assumption is supported by the fluctuation in hemoglobin concentration, where a slight decrease on day 7 was followed by an increase in values on day 16 and a further increase on day 28 in both groups.

Thrombocyte (platelet) counts agree with the RBC counts and suggest that blood loss may have been greater in the fish-skin-treated mice as a significant increase in platelet counts was recorded on day 16 relative to day zero and day 7 baselines. The counts normalized and were identical between the controls and fish-skin-treated mice on day 28 (Figure 4). It is possible that the adhesive nature of the HCB bandage facilitated better hemostasis and prevented blood loss, while fish skin offered no advantage in hemostasis. Nevertheless, blood loss was not significant, and no mice exhibited signs of anemia at any time point in the study.

Blood vessel formation is crucial to transport nutrients, immune cells, and oxygen to facilitate wound healing. According to the most recent understanding of the burn model zones [52], the tissue from the outer two zones of the burn wound be salvageable if vasculature is revived quickly [53]. In the present study, histology sections revealed more vessel formation in the fish-skin-treated group at day 16 than in controls (Figure 5 and Figure 6). The earlier vessel formation in fish-skin-treated mice aligns with reports by Ibrahim et al. [35], who showed a similar trend of vascularization on day 14 of metacarpal wounds in donkeys treated with fish skin compared to metacarpal wounds dressed with sterile, non-adherent dressing pads. The qPCR analysis agreed with the histological observations. It showed significantly greater (two-fold) expression of VEGF on day 16 in fish-skin-treated groups compared to controls, reflecting the beneficial effect of fish skin on angiogenesis. Lastly, the thicknesses of the skin at the interface of the burn with healthy skin were significantly thicker in fish-skin-treated mice at the 28 d time point, suggestive of a thicker layer of granulation tissue in these animals.

The unpublished data from clinical treatments of veterinary patients with tilapia fish skin during recent fires in California suggested that fish skin therapy for burns had an immediate pain-mediating effect. For example, bears with burned paws could walk after fish skin application but not when wounds were treated conservatively with bandaging. We therefore attempted to explore if fish skin treatment of burns on mice would have some effects on pain mediators or their receptors. Close observation of experimental animals and unchanged body weight throughout the study did not support a difference in pain sensation between experimental groups. Our PCR exploration of pain-associated gene expression of substance P, bradykinin receptor 2, neuropeptide Y, and calcitonin gene-related protein did not reveal significant differences between treatment groups, and thus neither confirm nor corroborate clinical observations of pain relief (Figure 7). Further, more mechanistic investigations on the fish skin’s effect on pain sensation using specific agonists and antagonists to determine if fish skin has any benefit in pain mitigation would be needed to elucidate this aspect.

Fish-skin-treated mice had nearly two times more expression of defensin (Figure 7) in comparison to controls at all time points, albeit not statistically significant. Clinical studies performed on human patients with burns found that defensin expression is reduced in burn wounds [54]. The increased expression of defensin in fish-skin-treated mice may suggest that fish skin facilitated the secretion of this peptide. Although no bacterial infection was detected in any of the groups in this study, further studies with controlled bacterial infections may be beneficial to further investigate the relevance of this finding. A similar but less-pronounced trend was detected in the expression of cathelicidin antimicrobial peptide in this study. Albeit not significantly different from controls, cathelicidin antimicrobial peptide expression trended higher in fish-skin-treated mice. Provided the importance of this antimicrobial peptide in controlling bacterial infection in a knockout mouse model [55], further study would be beneficial.

Proteomic analysis of fish skin revealed 22 unidentified nonstructural peptides (Figure 8). Upon blasting these sequences against the database, identity with mammalian antimicrobial, tissue repair, and anti-inflammatory peptides was detected. Specifically, the FHL2 peptide was identified. This peptide is involved in platelet activation, pathogen clearance, cytokine regulation, and cell proliferation [56]. According to Wixler et al., the failure of tissue repair is always accompanied by the suppression of FHL2 [56]. Other nonstructural peptides relevant to this study, such as apolipoprotein, periostin, hemopexin, and gelsolin, were also detected. Although the functions of many of these peptides are still not fully understood, all of them are mentioned in the context of wound healing. For example, apolipoproteins are lipoproteins with many metabolism-related functions. Specifically, Apolipoprotein E was shown to correct delayed wound healing in Apolipoprotein E-deficient mice [57], and Apolipoprotein A could suppress the growth of Escherichia coli and Klebsiella pneumoniae [58]. Periostin was explored in a mouse wound-repair study, where signaling through integrin molecules was shown to promote the proliferation and migration of fibroblasts [59]. Another study detected overexpression of this peptide in the pericytes of burned skin, and its function was related to scar formation and fibrosis [60]. Gelsolin function is related to actin modulation and clearance after tissue injury [61]. Administration of recombinant gelsolin was reported to cause antioxidant activity in a concentration-dependent manner in murine fibroblasts [62]. Also, decreased gelsolin plasma concentration was associated with increasing burn sizes and the severity of sepsis in human patients [63]. Undeniably, more work needs to be performed to identify fish skin peptides with the most crucial biological functions relevant to burns and other types of wound repair.

In conclusion, the data from this study uncover some of the potential key contributors to fish skin’s healing properties and the advantages of using fish skin in the treatment of burn wounds. This study’s most significant finding is increased angiogenesis with fish skin treatment. The limitation of this study was the inability to objectively assess the wound closure efficacy due to the innate biological constraints of the model. This limitation may have obscured the additional advantages of this treatment and therefore warrants exploring the wound-closure speed in a different animal model, such as a pig. It was also difficult to maintain equal age-matched male-to-female distribution across the treatment groups due to adverse responses to the burns by the smaller females (<25 g). Nevertheless, females who completed the study were not statistically different from males in any outcome parameters. More studies need to be performed to identify specific components of fish skin and their mechanistic role in wound healing. Also, application to other types of wounds, such as diabetic wounds, bacterial wounds, antibiotic-resistant bacterial skin infections, or pressure sores, would be interesting. Moreover, combining fish skin with stem cell treatment would be an exciting direction to explore.

## Figures and Tables

**Table 1:** QRT-PCR primer sets.

Target Gene	Forward Primer 5′→3′	Reverse Primer 5′→3′	Reference
Cathelicidin antimicrobial peptide (CRAMP)	CCTAGACACCAATCTCTACC	GTCTCCTTCACTCGGAACC	[14]
β-defensin 1	CCAGATGGAGCCAGGTGTTG	AGCTGGAGCGGAGACAGAATCC	[15]
Substance P = Protachykinin-1 (Tac1)	CCAGATCTCTCACAAAAGGC	TTTCGTAGTTCTGCATCGCGC	[16]
Bradykinin receptor B1 (BDKRB1)	TGGAGTTGAACGTTTTGGGTTT	GTGAGGATCAGCCCCATTGT	[17]
Bradykinin receptor B2 (BDKRB2)	CTGGGTGTTTGGAGAGGTGT	ACGAGCATCAGGAAGCAGAT	[18]
Calcitonin gene-related protein (CGRP)	GGACTTGGAGACAAACCACCA	GAGAGCAACCAGAGAGGAACTACA	[19]
Neuropeptide Y	AGGCTTGAAGACCCTTCCAT	ACAGGCAGACTGGTTTCAGG	[20]
Vascular endothelial growth factor (VEGF)	GCAGAAGTCCCATGAAGTGAT	GTCTCAATTGGACGGCAGTAG	[21]
Fibroblast growth factor (FGF2)	GTCACGGAAATACTCCAGTTGGT	CCCGTTTTGGATCCGAGTTT	[21]
GAPDH	CATCACTGCCACCCAGAAGACTG	ATGCCAGTGAGCTTCCCGTTCAG	[21]
Interleukin 6 (IL-6)	CAAAGCCAGAGTCCTTCAGA	GATGGTCTTGGTCCTTAGCC	[22]
Inducible nitric oxide synthase (iNOS)	CAG CTG GGC TGT ACA AAC CTT	CAT TGG AAG TGA AGC GGT TCG	
Neuronal nitric oxide synthase (nNOS)	ACTGACACCCTGCACCTGAAGA	GGAAGTCAGAAGATGTCCGCAC	

**Table 2:** Survival outcomes.

Group Number	Treatment Plan	Male	Female	Total
1	FS/16	5	4	9
2	CR/16	5	3	8
3	FS/28	4	4	8
4	CR/28	5	5	10

CR = control; FS = fish skin; M = male; F = female; 16 = euthanized 16 days post-burn; 28 = euthanized 28 days post-burn. Table 2 reflects the distribution of male and female mice in each experimental group.

## Data Availability

The data to support these results and required to reproduce these findings are available to download from https://data.mendeley.com/datasets/hssv66g4kb/4, accessed on 14 September 2023.

## Supplementary Materials

The following supporting information can be downloaded at: https://www.mdpi.com/article/10.3390/jfb14100512/s1, Figure S1: Schematic of tilapia skin preparation; Figure S2: Percent Leukocyte Blood Analysis; Figure S3: The thickness of the skin at the burn-healthy skin interface measure on the histological sections; Video S1: Debridement procedure.
