# Supplementary material for: Tilapia Fish Skin Treatment of Third-Degree Skin Burns in Murine Model
Source: J Funct Biomater. 2023 Oct 11;14(10):512. doi: 10.3390/jfb14100512 (PMC10607444; doi:10.3390/jfb14100512)
Supplement: Supplementary file 1 [file jfb-14-00512-s001.zip › supplementary-Figures.pdf]

Journal of *Functional Biomaterials*

MDPI

Supplementary Materials

Tilapia Fish Skin Treatment of Third-Degree Skin Burns in Murine Model

Carissa Garrity [1], Christina Garcia-Rovetta [1], Iris Rivas [1], Ubaldo Delatorre [1], Alice Wong [2], Dietmar Kültz [3], Jamie Peyton [4], Boaz Arzi [5] and Natalia Vapniarsky [1,*]

[1] Department of Pathology, Microbiology, and Immunology, University of California, Davis, CA, 95616, USA; crgarrity@ucdavis.edu (C.G.); ccgarciarovetta@ucdavis.edu (C.G.-R.); irlrivas@ucdavis.edu (I.R.); udelatorre@ucdavis.edu (U.D.)

[2] Department of Anatomy, Physiology and Cell Biology, School of Veterinary Medicine, University of California, Davis, CA, 95616, USA; awong@ucdavis.edu

[3] Department of Animal Sciences and Coastal & Marine Sciences Institute, Davis, CA, 95616, USA; dkueltz@ucdavis.edu

[4] One Health Institute, School of Veterinary Medicine, University of California, Davis, CA, 95616, USA; jlpeytondvm@icloud.com

[5] Department of Surgical and Radiological Sciences, School of Veterinary Medicine, University of California, Davis, CA, 95616, USA; barzi@ucdavis.edu

* Correspondence: vapniarsky@ucdavis.edu

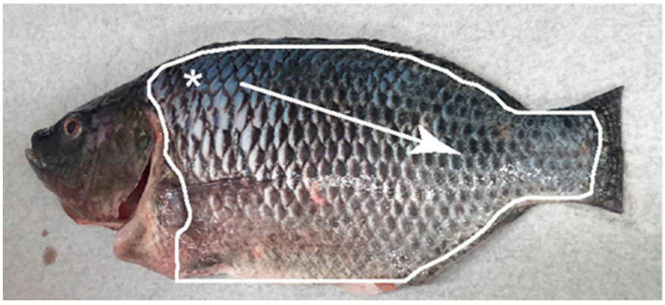

Figure S1. Schematic of tilapia skin preparation. The skin was cut along the solid lines and then pulled in the direction of the arrow from the dorso-cranial corner (*). The procedure was repeated on opposite side.

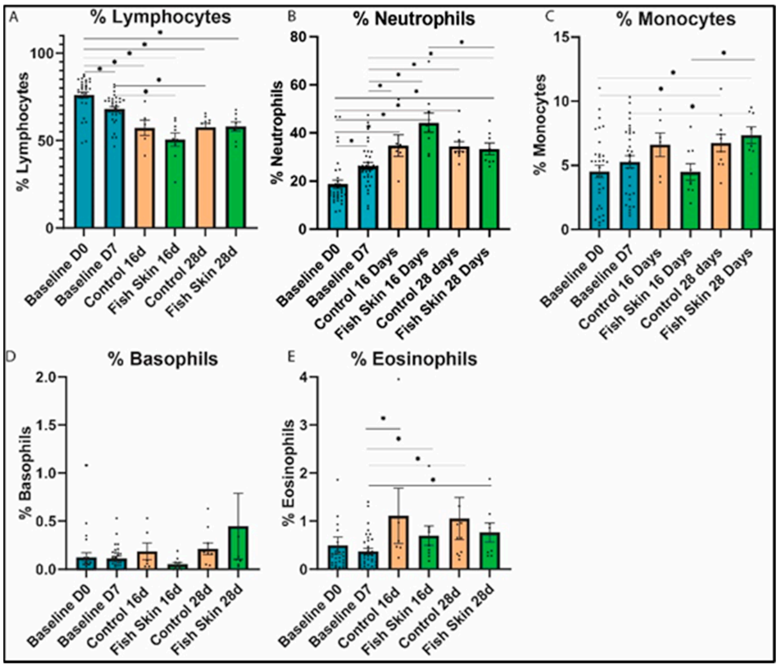

Figure S2. Percent Leukocyte Blood Analysis. (**A**) Percent lymphocytes at days 0 ,7, 16, and 28. (**B**) Percent neutrophils at days 0, 7, 16, and 28. (**C**) Percent monocytes at days 0, 7, 16, and 28. (**D**) Percent basophils at days 0, 7, 16, and 28. (**E**) Percent eosinophils at days 0, 7, 16, and 28. $P \leq 0.05$ (*). Baseline groups represent the average parameters of all mice, regardless of treatment. All mice were treated the same until day 7.

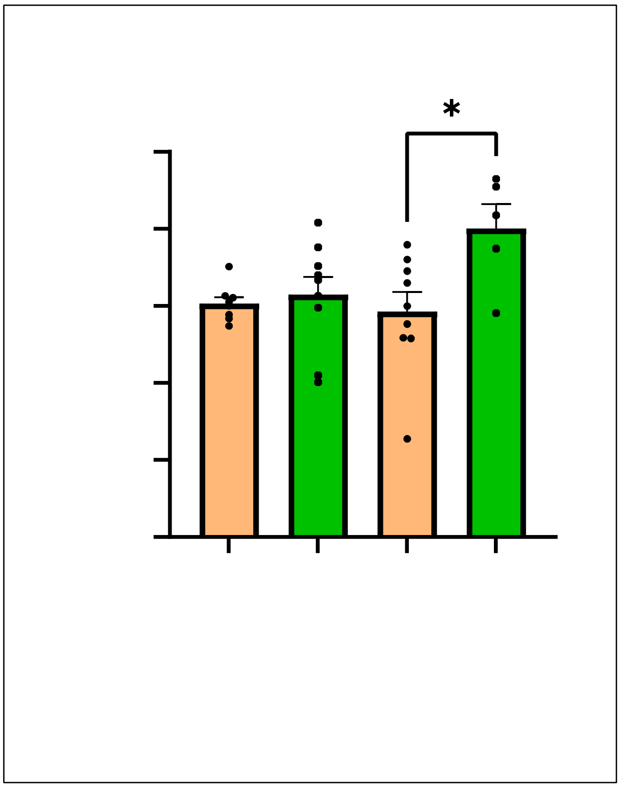

Figure S3. The thickness of the skin at the burn-healthy skin interface measure on the histological sections. $P \leq 0.05$ (*). Error bars represent standard deviation. .
